# Editorial: In celebration of women in molecular and cellular reproduction

**DOI:** 10.3389/fcell.2024.1402553

**Published:** 2024-05-01

**Authors:** Vanina Gabriela Da Ros, Dolores Busso, Patricia S. Cuasnicú

**Affiliations:** ^1^ Instituto de Biología y Medicina Experimental (IBYME), Fundación IBYME, Consejo Nacional de Investigaciones Científicas y Técnicas (CONICET), Buenos Aires, Argentina; ^2^ Biomedical Research and Innovation Center, Universidad de los Andes, Santiago, Chile

**Keywords:** women, reproductive tract, sperm, egg, fertilization, infertility

Throughout history, women have made significant contributions to research but have often faced challenges and barriers to being recognized and valued on equal terms with their male counterparts. In this regard, the role of women in scientific publications is a crucial topic in the academic and scientific realm. Although the proportion of women co-authoring scientific papers has increased over the past 2 decades, inequalities still exist in representation and recognition reflected in their position as first or last authors, citation index and the negative correlation between journal rank and the percentage of women co-authors (Holman et al., 2018). Thus, it is crucial to highlight and celebrate the achievements of women in science through visibility in scientific publications, and scientific journals play a crucial role in ensuring that women’s contributions are valued and adequately recognized.

As a way to highlight and celebrate the achievements of women in science, Frontiers in Cell and Developmental Biology is proud to offer this Research Topic to promote the work of women scientists across the molecular and cellular reproduction field. Across the globe, female scientists lead pioneering investigations aimed at unraveling the intricate mechanisms governing fertility regulation and preservation and at devising innovative strategies to overcome infertility-related hurdles. This Research Topic includes eleven articles in which all first or corresponding authors identified as women. Contributors are from different continents, with authors from North America (i.e., Canada and USA), South America (i.e., Argentina, Uruguay), Europe (Lituania, Hungary and Spain) and Asia (i.e., China); five of these papers address sperm and male fertility topics whereas the remaining six cover different aspects of egg, embryos and female fertility.

Among the papers describing experimental results in the male, Romarowski et al. seek to uncover novel metabolic pathways underlying sperm function in mice. Their study investigates energy restriction and recovery (SER), a novel method that has shown promising results in enhancing fertilization rates and embryo development following *in vitro* fertilization (IVF) in mice. By subjecting mouse sperm to SER and analyzing metabolism and capacitation-associated signaling, Romarowski et al. gain insights into the increased energy consumption rates and metabolic adaptations occurring within capacitating sperm. Such findings not only deepen our understanding of sperm physiology, but also hold implications for improving assisted reproductive techniques and fertility outcomes.

Vaquer et al. investigate the intricate signaling cascades governing human sperm acrosomal exocytosis, a crucial pre-fertilization event regulated by calcium dynamics. Their research focuses on the role of sphingolipids, particularly ceramide 1-phosphate and ceramide kinase, in orchestrating the intricate interplay between calcium release and progesterone-induced reactions essential for sperm competence and fertilization success. Through their experimentation and data analysis, Vaquer and her team unveil the pivotal involvement of ceramide signaling in mediating acrosomal exocytosis, thereby providing valuable insights into potential targets for therapeutic interventions to enhance fertility.

Toledo-Guardiola et al. delve into the role of seminal plasma in modulating sperm function within the female reproductive tract. Their study investigates how different seminal ejaculated fractions impact the protein cargo of extracellular vesicles in pigs, offering insights into the molecular mechanisms underlying successful fertilization and embryo development. By elucidating the interactions between seminal plasma components and female reproductive tissues, Toledo-Guardiola et al. aim to contribute to novel potential therapeutic strategies to enhance reproductive success.

Expanding the horizon of reproductive research, Guazzone and Lustig describe in their review the implications of immune dysregulation within the testicular microenvironment for male infertility. While the association between infectious diseases and autoimmune infertility has been well-established, the link between non-infectious pathological conditions and autoimmunity remains relatively unexplored. Guazzone and Lustig describe novel aspects of testicular pathologies and inflammation, highlighting the intricate interplay of immune factors in modulating testicular autoimmunity and paving the way for targeted therapeutic interventions to alleviate some cases of male infertility.

In the last contribution focusing on the male Kimelman et al., tackle the issue of fertility preservation in male cancer patients. Through a descriptive, cross-sectional study, Kimelman evaluate the fertility status of male cancer survivors who underwent sperm cryopreservation. Their findings not only provide valuable insights into counseling practices and reproductive outcomes post-cryopreservation but also underscore the importance of tailored health policies for addressing the reproductive needs of male cancer survivors.

On the female side of research on reproductive biology in this issue Marcela Michaut and her group (Klinsky et al.) use mouse gametes to shed light on the molecular mechanisms regulating the egg cortical reaction, a critical event triggered by fertilization. Their study explores the feasibility of utilizing cell-penetrating peptides as a non-invasive and efficient approach for studying the cortical reaction in mouse oocytes, a strategy that may accelerate research in this field.

Joo et al. explore the relationship between embryonic morphological parameters and their viability. Their study reveals a positive correlation between the presence of cytoplasmic strings and embryo viability, suggesting the potential utility of this parameter for selecting embryos with higher implantation potential. By offering a non-invasive method for assessing embryo quality, Joo et al. findings hold promise for improving embryo selection techniques and enhancing fertility outcomes in assisted reproductive procedures.

Potential mechanisms explaining female infertility due to known and unknown causes, and novel strategies developed to alleviate this condition, are addressed by four papers in this Research Topic. Geng et al. propose a novel therapeutic strategy for primary ovarian insufficiency (POI), a condition characterized by the premature depletion of ovarian follicles and infertility in young women. Their mini-review explores the potential use of stem cell-derived extracellular vesicles (EVs) as a remedy for POI. They describe studies that demonstrate the ability of EVs to promote follicular growth, reduce atresia, and restore hormone levels in animal models. The work of Geng et al. work holds significant implications for the development of novel therapeutic interventions to preserve fertility in women with POI.

Bausyte et al. investigate the therapeutic potential of endometrium-derived mesenchymal stem cells (hEnMSCs) for treating endometrial-factor-induced infertility. By employing hEnMSCs to treat mice with endometrial injury induced by mechanical damage or chemotherapy, Bausyte et al. demonstrate the ability of these stem cells to enhance endometrial restoration and improve fertility outcomes, offering a promising new approach for treating infertility associated with endometrial factors and, thus, hope to individuals struggling with reproductive challenges.

Luo et al. analyze the molecular mechanisms underlying recurrent pregnancy loss (RPL), a devastating condition that affects many couples worldwide. By investigating the role of D3 in mediating BMP2-induced downregulation of ICAM1 expression in human endometrial stromal and decidual cells, Luo et al. uncover novel factors and regulators implicated in RPL pathology, contributing to the development of targeted therapeutic interventions to mitigate this reproductive disorder.

Finally, Vaigauskaitė-Mažeikienė et al. study unexplained infertility, a perplexing condition affecting a significant proportion of infertile couples. By analyzing fertilization outcomes and gene expression patterns in the endometrium and follicular fluid of unexplained infertile couples, Vaigauskaitė-Mažeikienė et al. provide valuable clinical and molecular insights that pave the way for developing targeted therapeutic interventions for patients with idiopathic infertility.

In conclusion, the contributions of this Research Topic underscore the remarkable progress being made by women in the field of reproductive biology, from unraveling the intricate molecular mechanisms governing sperm function and embryo development to exploring innovative therapeutic strategies for addressing infertility-related challenges. By combining cutting-edge scientific techniques with molecular and clinical insights, these and other female researchers around the world lead the generation of knowledge to understand reproductive processes that can be translated into strategies to overcome the barriers to fertility and reproductive health.
